# Unloading of Right Ventricle and Clinical Improvement after Ultrasound-Accelerated Thrombolysis in Patients with Submassive Pulmonary Embolism

**DOI:** 10.1155/2014/297951

**Published:** 2014-07-01

**Authors:** Sachin Kumar Amruthlal Jain, Brijesh Patel, Wadie David, Ayad Jazrawi, Patrick Alexander

**Affiliations:** ^1^Department of Cardiology, Providence Hospital and Medical Center, 16001 W Nine Mile Road, Southfield, MI 48075, USA; ^2^Department of Internal Medicine, Providence Hospital and Medical Center, 16001 W Nine Mile Road, Southfield, MI 48075, USA

## Abstract

Acute pulmonary embolism (PE) can be devastating. It is classified into three categories based on clinical scenario, elevated biomarkers, radiographic or echocardiographic features of right ventricular strain, and hemodynamic instability. Submassive PE is diagnosed when a patient has elevated biomarkers, CT-scan, or echocardiogram showing right ventricular strain and no signs of hemodynamic compromise. Thromboemboli in the acute setting increase pulmonary vascular resistance by obstruction and vasoconstriction, resulting in pulmonary hypertension. This, further, deteriorates symptoms and hemodynamic status. Studies have shown that elevated biomarkers and right ventricular (RV) dysfunction have been associated with increased risk of mortality. Therefore, aggressive treatment is necessary to “unload” right ventricle. The treatment of submassive PE with thrombolysis is controversial, though recent data have favored thrombolysis over conventional anticoagulants in acute setting. The most feared complication of systemic thrombolysis is intracranial or major bleeding. To circumvent this problem, a newer and safer approach is sought. Ultrasound-accelerated thrombolysis is a relatively newer and safer approach that requires local administration of thrombolytic agents. Herein, we report a case series of five patients who underwent ultrasound-accelerated thrombolysis with notable improvement in symptoms and right ventricular function.

## 1. Introduction

Pulmonary embolism (PE) and deep venous thrombosis (DVT) are a major cause of morbidity and mortality in the United States and are the leading cause of death in hospitals today. Pulmonary embolism is a blood clot which originates in the deep venous system of the lower exterminates and travels to the lungs where it lodges in the main pulmonary artery or surrounding branches. The result is an obstruction of blood flow that ordinarily would be oxygenated and returned to the systemic circulation. Given the nature of the venous system blood flow, there is low pressure and slow moving increasing the probability of formation of blood clot [1]. Pulmonary embolism or venous thrombosis is a devastating diagnosis and is referred to as a silent killer. It affects over a million people every year with 100,000 to 200,000 of these cases fatal. Early diagnosis is the key to improved survival rates. Of the fatal cases, 20% die before their initial diagnosis or days later [2]. Once the diagnosis of PE is made, it should be classified further into one of three categories: (1) low-risk PE, (2) submassive PE, and (3) massive PE. The diagnosis of low-risk, submassive, and massive PE is made based on clinical presentation, abnormal biomarkers, echocardiography/imaging evidence, and hemodynamic instability.

For each category, the management significantly differs. A submassive PE is diagnosed when there is a characteristic of an embolus/emboli causing right ventricular strain without significant hypotension (systolic blood pressure <90 mmHg) and elevated biomarkers. When there is an evidence of shock (systolic blood pressure less than 90 mmHg), the patient has massive PE [3]. Thrombolytic therapy is recommended for patients with massive PE (assuming no contraindications); however, the use of thrombolytic drug is controversial in submassive PE [4]. Even though it is used with the best intention in such patient, the risk of bleeding should not be overlooked. Thrombolytic agents have been shown to increase risk of major bleeding, nonmajor bleed, and intracranial bleeding compared to heparin [5]. Therefore, alternative strategies are developed. An ultrasound-accelerated thrombolysis (UAT) is a relatively newer strategy that is intended to minimize risk of bleeding and improve outcomes. The UAT uses high-frequency, low power ultrasound wave that disrupts fibrin strands and makes the clot more permeable to a fibrinolytic agent. The fibrinolytic agent is delivered directly into the pulmonary artery along with intravenous anticoagulant. Once the fibrinolytic agent binds to the clot, it does not migrate into circulatory system [6]. The early goals of the treatment are preventing the progression of clot burden, improving hemodynamic status, and relief from the symptoms and long-term complications such as chronic thromboembolic pulmonary hypertension (CTEPH). We believe that UAT could be effective against acute burden on the right ventricle, even in the cases of acute on chronic pulmonary embolism, and will improve hemodynamic status and relief of symptoms. Hopefully, this will prevent CTEPH as systemic anticoagulants will take over. We report a case series of five cases that were treated with UAT demonstrating clinical improvement in the acute setting. All five patients gave informed consent before treating them with this alternative approach.

## 2. Case 1

**Figure 1:**
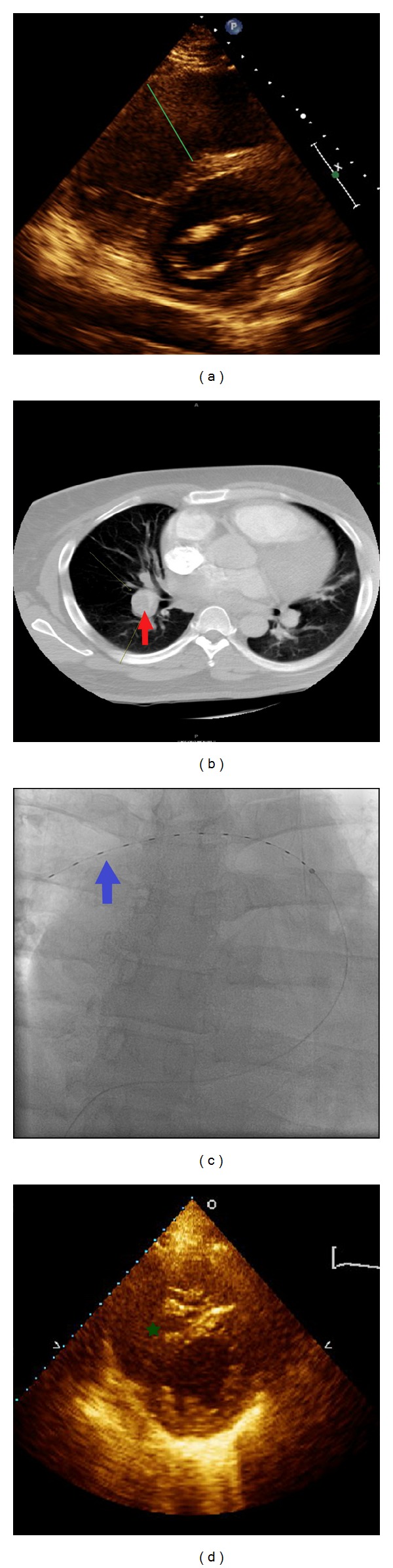
(a) The parasternal short axis view from echocardiography shows dilatation of the right ventricle (green line). (b) The CT-thorax with contrast filling defect in the right main pulmonary artery confirms the diagnosis of pulmonary embolism. (c) The fluoroscopy illustrates the placement of ultrasound-accelerated thrombolysis catheter in the right main pulmonary artery. (d) The improvement in right ventricular function after UAT (green asterisk).

A 38-year-old African American male was sent to the hospital by his primary care physician office after the patient complained of progressively worsening shortness of breath over the past two days. The patient has medical history of asthma and gout, but he denied this episode as his typical asthma attack. He stated that the symptoms worsen during night. Additionally, he complained of 1-month history of left lower extremity swelling after he had 9-10 hours long, nonstop road trip. He denied chest pain, palpitations, or lightheadedness. His initial vital signs were respiratory rate of 25 breaths per minute, blood pressure of 151/111 mmHg, heart rate of 108 beats per minute, oxygen saturation of 94% on room air, temperature of 97.7 Fahrenheit, and body mass index of 44. The patient's physical exam was remarkable for tachycardia, S3 gallop, and slight wheezing. His electrocardiogram showed sinus tachycardia, S1Q3T3 pattern, and incomplete right bundle branch block. His initial troponin, Pro-BNP, and D-Dimer were 1.09 ng/mL (nomral: 0–0.10 ng/mL), 6853 pg/mL (normal: 50–92 pg/mL), and 4060 ng/mL (normal: 200–232 ng/mL), respectively. The patient underwent CT-thorax, which showed emboli within the right lower and middle lobe arteries. Also, there was a 41 mm dilatation of the main pulmonary artery, cardiomegaly with small pericardial effusion and mild pulmonary edema. His lower extremity ultrasound with Doppler showed acute-to-subacute occlusive thrombus extending into the left common femoral vein. His echocardiography showed right ventricular enlargement with right-to-left ventricle ratio (RV/LV) greater than 1, dyskinesia of the RV (Figure 1), and right ventricle systolic pressure (RVSP) of 75 mmHg. He also had mild left ventricular enlargement and ejection fraction of 20%.

Due to the significant right ventricular strain and elevated troponin, a submassive PE was diagnosed. The patient underwent UAT (1 mg/hr of alteplase was delivered for 12 hours via the catheter in Figure 1) in the right pulmonary artery and he continued to receive intravenous heparin; he was medically managed under Intensive Care Unit care. As his hospital course progressed, the patient became asymptomatic with rapid improvement in next two days. He could ambulate without the need for oxygen supplement. An echocardiogram prior to discharge showed improvement in the right ventricular function and RV/LV1. At this time, the RVSP was 50 mmHg. He was discharged home with Coumadin. His follow-up CT scan at 6 months demonstrated persistent emboli in the right lower pulmonary artery despite the oral anticoagulant. The patient was referred for surgical thrombectomy. Refer to Table 1 for data summary.

## 3. Case 2

**Figure 2:**
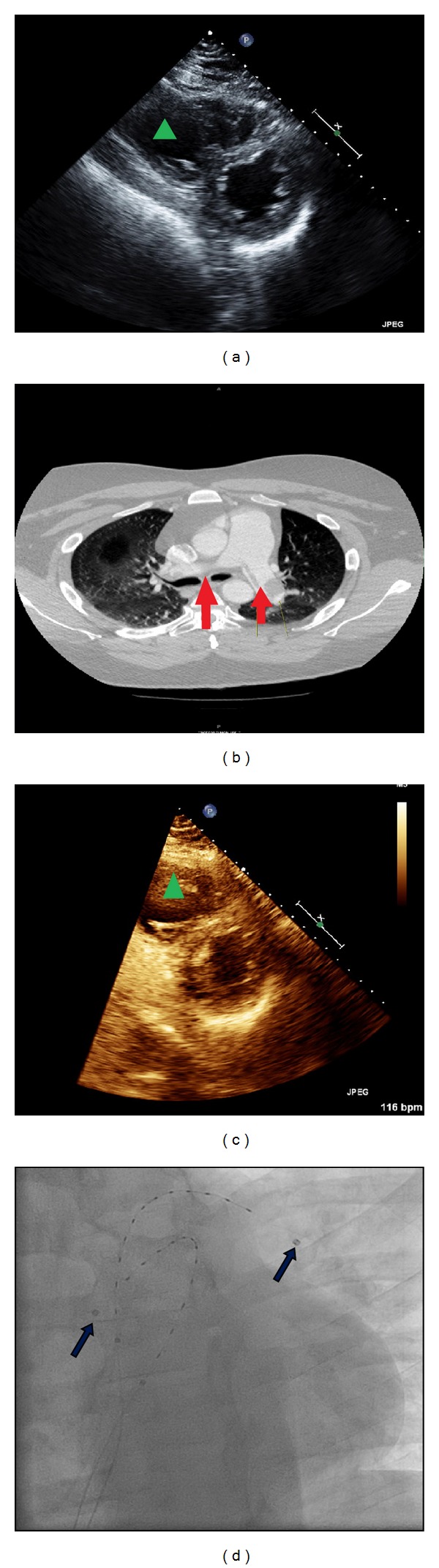
(a) The green triangle is located in the markedly dilated right ventricle. (b) The red arrows point at saddle emboli. (c) The echocardiogram shows improvement in the size of right ventricle. (d) The fluoroscopy confirms the placement of the UAT catheter in both main pulmonary arteries (blue arrows).

A 40-year-old African American male came to our hospital after he had undergone a syncopal episode. His past medical history includes deep vein thrombosis, chronic lower extremity lymphedema, and diabetes. The patient denied trauma, recent travelling, and sedentary lifestyle. He also denies history of hypercoagulable state. Upon arrival, his vital signs were respiratory rate of 30 breaths per minute, blood pressure of 124/92 mmHg, heart rate of 125 beats per minute, oxygen saturation of 98% on a nonrebreather mask, and temperature of 97.7 Fahrenheit. His physical exam was remarkable for anxious middle-aged man with tachycardia, jugular venous pressure of 15 cm, and venous stasis in the lower extremities. The electrocardiogram shows tachycardia, S1Q3T3 pattern, and poor R-wave progression in the anterior precordial leads. An initial laboratory workup revealed troponin level of 0.02 ng/mL (normal: 0–0.10 ng/mL), pro-BNP of 149 pg/mL (normal: 50–92 pg/mL), and D-Dimer at 5000 ng/mL (normal: 200–232 ng/mL). He underwent CT-thorax which showed extensive thrombus in both distal main pulmonary arteries with small component of main pulmonary artery saddle thrombus (Figure 2). The right lower extremity ultrasound with Doppler showed occlusive thrombus extending from the right popliteal vein to right common femoral vein.

An emergent echocardiogram was obtained, which showed marked dilatation of the right ventricle, RV/LV > 1, and severe right ventricular global hypokinesis (Figure 2). The RVSP was 70 mmHg. A submassive PE was diagnosed and the patient was taken to cardiac catheterization lab for UAT catheter placement; heparin and 1 mg/hr of alteplase were delivered via each catheter for 10 hours. Ten days later, a repeat transthoracic echocardiogram showed normal ejection fraction, RV/LV < 1, and minimal paradoxical septal motion (Figure 2) and RVSP of 62 mmHg. The patient no longer complained of dyspnea or lightheadedness. The patient was sent home on rivaroxaban and other home medications. Refer to Table 1 for data summary.

## 4. Case 3

**Figure 3:**
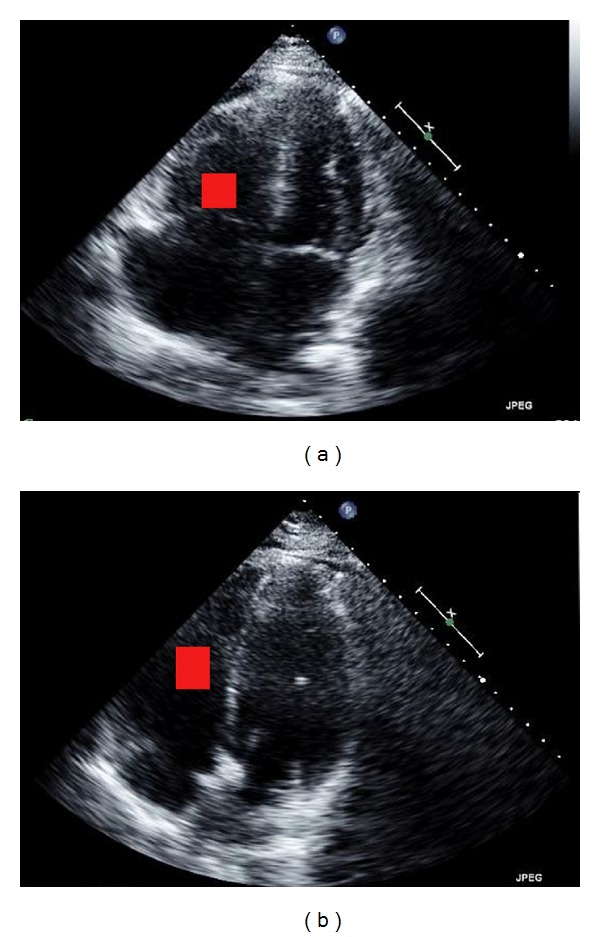
(a) The red square is in the right ventricle that is markedly dilated compared to left ventricle dimension. (b) The image shows the decrease in size of the right ventricle after the UAT (red square).

A 38-year-old African American female went to a community hospital for difficulty in breathing. A week before, she had an acute onset of difficulty in breathing, which was associated with occasional lightheadedness. Later, she had difficulty with performing her activity of daily leaving due to the shortness of breath. The patient has history of iron deficiency anemia and depression. She was on birth control pills, which she had been taking for the past 18 years. She is neither smoker nor substance abuser. At the time of presentation, she denied chest pain, lightheadedness, diaphoresis, headache, or dizziness. The initial vitals were blood pressure of 129/49 mmHg, respiratory rate of 15 breaths per minute, pulse rate of 75 beats per minute, and oxygen saturation of 95% on room air. She was afebrile. The cardiopulmonary, neurologic, and musculoskeletal exam was unremarkable. The laboratory workup did not reveal any electrolytes abnormalities; however, her pro-BNP was 1193 pg/mL (normal: 50–92 pg/mL) and troponin was 0.02 ng/mL (normal: 0–0.10 ng/mL). An electrocardiogram showed normal sinus rhythm with T-wave inversions in inferior leads and V1-V3 without ST-segment changes. A CT-thorax showed large bilateral pulmonary emboli in the pulmonary arteries. At this time, the patient was transferred to our hospital for further management. The echocardiogram revealed preserved left ventricular ejection fraction, hypokinetic RV with RV/LV >1 and severe pulmonary hypertension (right ventricular systolic pressure of 73 mmHg) (Figure 3). A lower extremity ultrasound with Doppler showed acute occlusive and nonocclusive deep vein thrombosis in left popliteal and right common femoral vein, respectively.

These findings were suggestive of submassive PE, which was treated with ultrasound accelerated thrombolysis. The right heart catheterization showed systolic pulmonary artery pressure of 51 mmHg. The catheters were placed in both pulmonary arteries and 13 mg of alteplase, along with heparin, was delivered over 16 hours with heparin drip. The patient tolerated the thrombolysis procedure without complication. The patient was discharged on oral rivaroxaban after the resolution of presenting symptoms. Three days later, the echocardiogram showed left ventricular ejection fraction of 65%, without the evidence of right ventricular strain (RV/LV < 1) or elevated right ventricle systolic pressure (Figure 3). The RVSP was 35 mmHg. Refer to Table 1 for data summary.

## 5. Case 4

**Figure 4:**
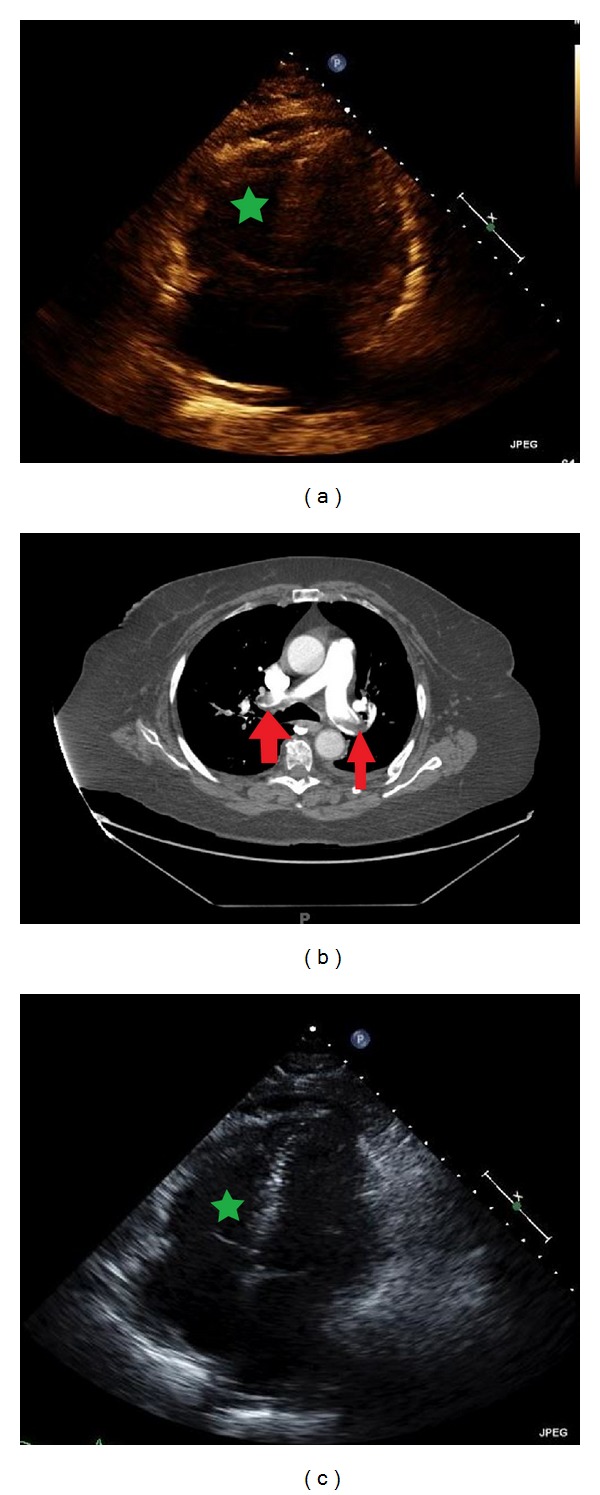
(a) The right ventricle is dilated and the septum is flattened (green asterisk). (b) The red arrows point at multiple bilateral emboli. (c) The image shows improvement in right ventricle dimension after the treatment.

A 77-year-old African American female came to our hospital complaining of shortness of breath. The patient was experiencing severe left-sided abdominal pain for the past two weeks. It was not associated with any alleviating or aggravating factors. On the day of presentation, she became lightheaded and experience shortness of breath. The discomfort was associated with two episodes of nonbloody emesis and left-sided pleuritic chest pain. She did not have any retrosternal chest pain, diaphoresis, or syncopal episode. She was, additionally, complaining of right lower leg cramping. Her medical history included hypertension, diabetes, and deep vein thrombosis, which was treated with an oral anticoagulant for six months. The initial vitals were blood pressure of 125/62 mmHg, heart rate of 92 beats per minute, respiratory rate of 18 breaths per minute, and oxygen saturation of 99% on room air; she was afebrile. The cardiopulmonary exam was unremarkable. The initial troponin level was 0.02 ng/mL (normal: 0–0.10 ng/mL), eight hours apart. The pro-BNP level was at 7511 pg/mL (normal: 50–92 pg/mL). An electrocardiogram showed normal sinus rhythm without significant findings. A CT-chest with contrast showed diffuse bilateral pulmonary emboli in the right and left main pulmonary arteries and straightening of the interventricular septum suggestive of right ventricular strain (Figure 4). The echocardiogram showed preserved ejection fraction, hypokinetic RV, mild pulmonary hypertension (RVSP of 49 mmHg), and RV/LV > 1 (Figure 4). The ultrasound with Doppler of the lower extremities did not show deep vein thrombosis.

The patient was diagnosed with submassive PE, and she was decided to be treated with UAT. The UAT catheters were placed in both arteries and local alteplase was administered for 12 mg with heparin over 12 hours after receiving 2 mg of bolus. There were no peri- or postprocedural complications. The next day, the patient experienced marked improvement in dyspnea. Next day, a repeated echocardiography showed preserved ejection fraction with mildly depressed right ventricular function with RV/LV <1 (Figure 4) and RVSP of 40 mmHg. The patient was discharged on oral rivaroxaban. Refer to Table 1 for data summary.

## 6. Case 5

**Figure 5:**
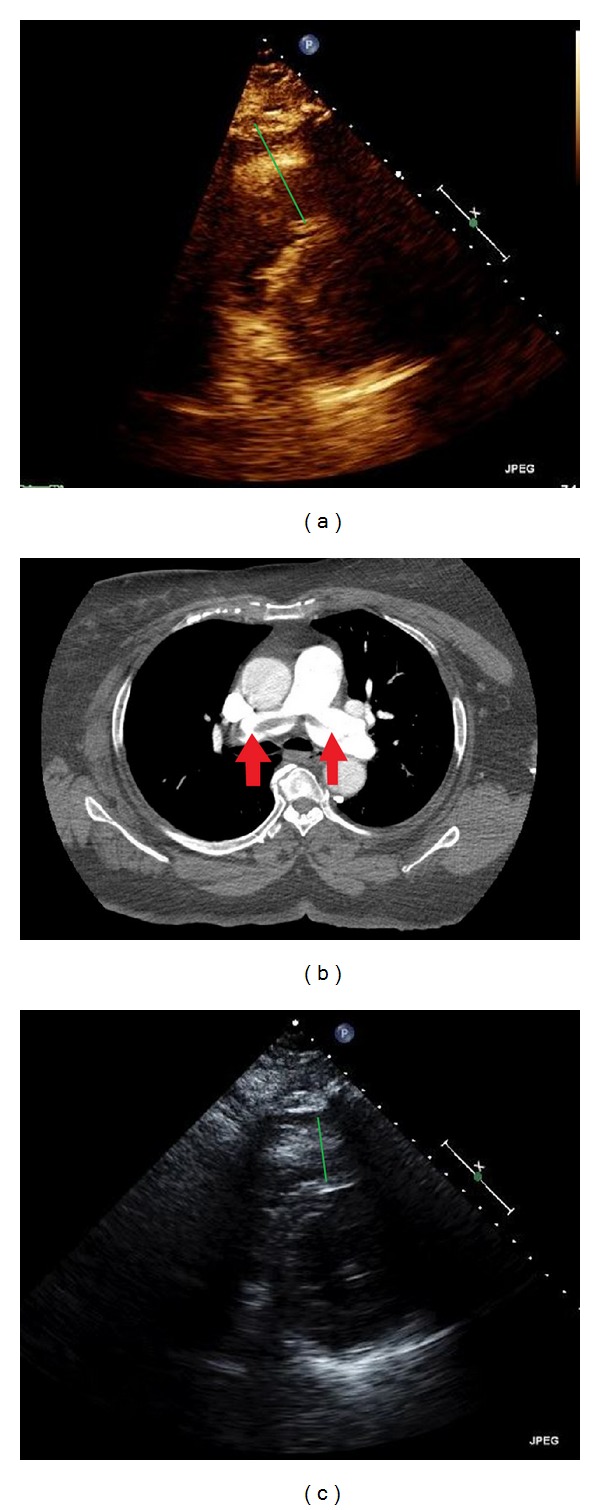
(a) The echocardiogram shows dilated right ventricle (green line). (b) The CT-scan shows an evidence of saddle emboli (red arrows). (c) There was a marked improvement in right ventricle size after the treatment (green line).

A 72-year-old African American female came to the hospital complaining of shortness of breath. The patient stated that the dyspnea started to progress over three days limiting her activity of daily living. The symptoms would worsen with exertion. She denied any chest pain, lightheadedness, diaphoresis, nausea, or vomiting. Her medical history includes hypertension, chronic obstructive lung disease, and dyslipidemia. At the time of presentation, the vital signs were blood pressure of 136/66 mmHg, heart rate of 75 beats per minute, respiratory rate of 18 breaths per minute, oxygen saturation of 95% on room air, and no fever. The cardiopulmonary, extremities, and neurologic exam was unremarkable. An electrocardiogram revealed normal sinus rhythm without significant abnormal findings. An initial laboratory workup showed pro-BNP level of 603 pg/mL (normal: 50–92 pg/mL), troponin of 0.02 ng/dL (0–0.10 ng/mL), and D-Dimer at 2072 ng/mL (normal: 200–232 ng/mL). A CT-scan of thorax with contrast revealed saddle pulmonary embolus of the right and left main pulmonary artery with extensive emboli (Figure 5). An emergent echocardiography showed normal ejection fraction with hypokinesia of right ventricular free wall. There was an evidence of moderate pulmonary hypertension (RVSP of 58 mmHg). Based on these findings, a submassive PE was diagnosed (Figure 5). The ultrasound with Doppler of the lower extremity showed no evidence of deep vein thrombosis.

For the treatment, the patient was taken for right heart catheterization for the placement of UAT in the main pulmonary arteries. The UAT catheters were placed in both pulmonary arteries, and heparin and 0.5 mg/hr of alteplase were delivered to each artery for 12 hours. There were no complications. Two days later, repeated echocardiogram showed marked improvement in right ventricle function (RV/LV < 1) and right ventricle systolic pressure (45 mmHg) (Figure 5). The patient denied exertional dyspnea and could ambulate without limitations. She was discharged to home with oral rivaroxaban. Refer to Table 1 for data summary.

## 7. Discussion

Once the diagnosis of pulmonary embolism is confirmed, the treatment strategies should be based on the classification of pulmonary embolism. A pulmonary embolism with hemodynamic compromise should be treated more aggressively with fibrinolytics (if there are no contraindications and acceptable bleeding risk) or surgical embolectomy [7]. In contrast, patients with PE and without any evidence of hemodynamic compromise or right ventricular strain are classified as low-risk PE [3].

A submassive PE is classified based on elevated cardiac biomarkers (e.g., Pro-BNP and troponin) and right ventricular strain without hemodynamic compromise [8]. Studies have shown that patients with right ventricular dysfunction, elevated troponin, and NT-pro-BNP levels have been associated with increased rate of mortality compared to baseline or normal values, respectively [9–11]. In acute setting of pulmonary embolism, increase in pulmonary vascular resistance results in elevated right ventricular systolic pressure up to 40 mmHg (nearly doubles from the baseline). Some patients with higher right ventricular systolic pressure than 40 mmHg may be attributable to coexisting pulmonary hypertension [12], including acute or chronic thrombus. The net effect is dilatation and dyskinesia of right ventricle, tricuspid regurgitation resulting in right ventricular failure. This leads to right ventricular ischemia/microinfarction (the cause of troponemia) and pressure overload (elevated pro-BNP) [13, 14]. The early compensation of reduced blood pressure from RV failure masks the impeding shock [12]. Therefore, patients with high-index of clinical suspicion for submassive PE should be evaluated with cardiac biomarker and imaging modality to assess the right ventricle. The RV strain is best assessed by emergent echocardiography. The characteristic findings include RV dyskinesia and dilatation (sometimes exceeding the dimensions of left ventricle), flattening of septum (the right ventricle appears like alphabet letter “D”), paradoxical movement of septum towards left ventricle, and pulmonary hypertension [8]. Bases on these findings, an aggressive treatment plan should be formulated to avoid fatal complications.

The role of fibrinolytics is controversial in such patients and anticoagulants are mainstay of treatment. The role of fibrinolytics in submassive PE has been assessed in MAPPET-3 trial which has shown reduced in-patient mortality and clinical deterioration requiring escalation of therapy in fibrinolytics plus heparin arm than heparin alone [15]. In another prospective trial, the use of fibrinolytics had advantage over heparin alone in reduction of pulmonary hypertension at 6-month follow-up [16]. A recently published trial (>1000 patients) has demonstrated that intermediate-risk PE patients (submassive PE) treated with fibrinolytics plus anticoagulant are less likely to have hemodynamic compromise than placebo group [17]. Despite the benefits of using fibrinolytics in submassive PE, the most feared complication is fatal bleeding (e.g., intracranial or gastrointestinal bleeding). Therefore, less harmful yet effective alternative should be sought; these include catheter-based, local administration of fibrinolytic agent, surgical embolectomy, and fragmentation or aspiration of the thrombus [8]. A surgical option is reserved for those who have contraindications or refractory to fibrinolytic agent [7].

Currently, ultrasound-accelerated thrombolysis (UAT) is an emerging option. It prevents hemolysis and mechanical fragmentation of the emboli, which are the complications of rheolytic-based mechanical fragmentation of the thrombus [6]. In comparison to systemic thrombolysis, UAT requires less fibrinolytic agents, thus, a fewer chances of fatal bleeding. The efficacy of UAT against anticoagulant therapy has been studied in ULTIMA trial. The study showed statistically significant reduction in right ventricular strain within 24 hours in UAT plus heparin versus heparin alone group without significant bleeding. In addition, there was marked reduction RV/LV ratio. The trial reported no major bleeding complications. The UAT was concluded to be superior to heparin alone [18]. Another trial that is evaluating the role of UAT in submassive and massive PE is under investigation. When there is persistent vascular obstruction and vasoconstriction, CTEPH could result. It has shown fibrinolytics to reduce frequency of CTEPH [19], though there is no published data on UAT and CTEPH. The limited available data have shown that UAT is safer and has effective modality in comparison to an anticoagulant alone. To our knowledge, there is no published data comparing UAT and other catheter-directed therapy or surgical embolectomy in submassive PE. Our cases suggest that ultrasound-accelerated thrombolysis can be effective in unloading of the right ventricle and decreasing pulmonary hypertension (decreasing right ventricle pressure load) in the acute setting without any bleeding complications from the fibrinolytic agent. Long-term anticoagulants and body hemostasis will take over and may help dissolve the clot completely.

## 8. Conclusion

The case series demonstrates that UAT is associated with clinical improvement in the acute clinical setting without major bleeding complications, and it adds to current literature. In patients with submassive PE and RVSP greater than 40 mmHg, UAT will relieve acute component. The long-term management should be based on available guideline.

## Figures and Tables

**Table 1:** Summary of the patients' data.

	Patient 1	Patient 2	Patient 3	Patient 4	Patient 5
Presenting symptoms	Dyspnea	Syncope	Dyspnea	Dyspnea	Dyspnea

Source of thrombus	DVT	DVT	DVT	Unknown	Unknown

Pro-BNP (pg/mL)	6853	149	1193	7511	603

Troponins (ng/mL)	1.09	0.02	0.02	0.02	0.02

D-Dimer (ng/mL)	4060	5000	N/A	N/A	2072

CT-scan findings	Right middle and lower lobe emboli	Saddle emboli	Bilateral emboli within main pulmonary arteries	Bilateral emboli within main pulmonary arteries	Saddle emboli

Echocardiogram (RVSP in mmHg)	Hypokinetic RV enlargement (75), RV/LV > 1	Markedly dilated and hypokinetic RV (70), RV/LV > 1	RV/LV > 1, severe pulmonary hypertension (73), and hypokinetic RV	Right ventricular strain, straightening of the septum (57), RV/LV > 1	Hypokinesia of right ventricular free wall (60), RV/LV > 1

Alteplase amount (per PA)	12	10	13	12	6

Follow-up echocardiogram (RVSP in mmHg)	Improved function of the right ventricle (50), RV/LV < 1	Mild RV dyskinesia (62), RV/LV < 1	No RV strain or pulmonary hypertension (35), RV/LV < 1	Mildly depressed RV function (40), RV/LV < 1	Resolution of RV hypokinesia (45), RV/LV < 1

Presenting symptoms at the time of discharge	Resolved	Resolved	Resolved	Resolved	Resolved

DVT = deep vein thrombosis; BNP = brain-natriuretic peptide; sPAP = systolic pulmonary artery pressure during right heart catheterization; RV = right ventricle; LV = left ventricle; PA = pulmonary artery; RVSP = right ventricular systolic pressure.
